# Peter McGuffin, PhD, FRCP, FRCPsych, FMedSci, CBE

**DOI:** 10.1192/bjb.2024.59

**Published:** 2024-10

**Authors:** Michael J. Owen

Formerly Director, MRC (Medical Research Council) Social Genetic & Developmental Psychiatry Research Centre, King's College, London

**Figure:**
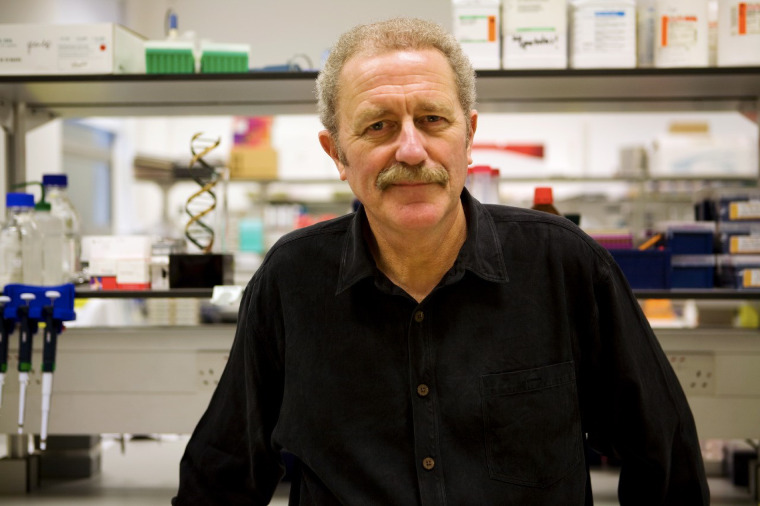

Peter McGuffin, who died on 30 January 2024, aged 74, was a pioneering psychiatric geneticist. He was able to demonstrate how genetic and non-genetic factors act together to predispose people to psychiatric disorders: so-called gene–environment interplay. He was also among the first to see the potential of the DNA revolution in understanding psychiatric disorders, and his work laid the foundations for the application of genomics to psychiatry. Working at the Institute of Psychiatry in London during the 1980s, Peter showed that adverse circumstances and genetic risk frequently combine to increase the likelihood of depression. These findings indicated that biological treatments such as antidepressants and approaches to prevention and treatment based on reducing exposure to or the impact of adverse circumstances could both be applicable to depressive illnesses.

He showed that adverse circumstances as well as depression tend to run in families. In subsequent work in Cardiff, he and his wife, Anne Farmer, an academic psychiatrist with whom he published many papers, demonstrated the complexity of the mechanisms involved, with adverse events reflecting a combination of a hazard-prone lifestyle, a propensity to over-perceive adversity and a tendency for adverse events to be overrepresented in relatives. This pointed to the need for a much more careful dissection of gene–environment interplay.

Psychiatric diagnoses are largely syndromic, based on clinical symptoms and signs rather than special investigations such as brain scans or blood tests. Nevertheless, it was widely believed that different diagnoses identified distinct conditions. Peter's work with twins in the 1990s challenged this conclusion when he showed that the genetic components of schizophrenia and bipolar disorder overlap, as well as there being components that are specific to each syndrome. Subsequently, he observed a similar pattern of shared and specific genetic components in bipolar disorder and major depression. These findings were among the first to point to the now widely held view that our current diagnostic approaches do not define distinct disorders and that we need better ways of defining severe mental illness.

Until the 1990s, research into the causes of childhood psychiatric conditions had focused largely on social and psychological factors. Peter undertook influential research, again using twin studies, that demonstrated the additional need to consider biological and particularly genetic factors. He showed that genes influence the occurrence of depressive symptoms in children, but that although the genetic effects are particularly important in adolescence, they are much less so in younger children. He also conducted some of the earliest studies demonstrating the heritability of attention-deficit hyperactivity disorder, paving the way for subsequent genomic work identifying specific genetic risk factors.

Born in Belfast, Peter was the eldest of three children of Martha Melba (*née* Burnison) and William Brown McGuffin, a merchant navy officer. When William was appointed as a Trinity House Pilot for the Port of Southampton in 1959, they moved to the Isle of Wight. From Sandown Grammar School, Peter went to medical school at the University of Leeds, where he met Anne Farmer. They married in 1972, the year that they graduated. In his subsequent training, he became interested in genetics and, while a junior doctor, together with Anne, carried out a genetic marker study of schizophrenia that suggested an association with the human leucocyte antigen system, a finding subsequently confirmed by genomic studies. Completing his training as a psychiatrist at the Maudsley Hospital, London, he was awarded a Medical Research Council (MRC) fellowship to study genetics and subsequently became an MRC senior clinical fellow at the Institute of Psychiatry (now the Institute of Psychiatry, Psychology and Neuroscience and part of King's College London).

In 1987, aged only 37, he was appointed to the chair of Psychological Medicine at the University of Wales College of Medicine in Cardiff (now part of Cardiff University). He held this position until 1998, when I succeeded him. During this period, he laid the foundations for Cardiff to become a centre of excellence in psychiatric genetics and trained several young researchers who would go on to make important contributions to this field. He also improved postgraduate psychiatric training across Wales, establishing an MSc in psychiatry and a subdepartment in North Wales. While in Cardiff, he was one of the founders of the International Society of Psychiatric Genetics and its second president. He was quick to see that the application of genomics would require large-scale international collaboration and established a European Science Foundation programme to bring together psychiatric genetics research across Europe in the 1980s and 1990s. This inspired a similar multicentre collaboration in the USA and laid the foundations for international collaborations that continue successfully to this day. One of the challenges faced by these international efforts was the need to establish diagnostic standardisation and reliability across the participating centres. Peter developed the computerised OPCRIT system, which showed good reliability across the European and US centres and remains widely used in genetic and epidemiological studies.

From Cardiff he went on to head the MRC Social Genetic & Developmental Psychiatry Research Centre (SGDP) in London. This was entirely appropriate, as his early work on depression had been a major stimulus for establishing this centre, whose aim is to integrate genetics with social and developmental research. He led the SGDP with great distinction, developing a supportive and nurturing environment that allowed its many stars to shine while also developing the careers of numerous students and junior scientists.

The SGDP expanded under his leadership, attaining international recognition for its excellence in multidisciplinary psychiatric research, and Peter successfully raised the funding for a new building to house it. His leadership and skills as an administrator were recognised by his appointment in 2007 as dean of the Institute of Psychiatry, a post he held for 3 years, during which he rescued the Institute of Psychiatry from a financial crisis. Through all this, he remained a productive and highly cited researcher, publishing more than 500 papers, with an h-index >100, and eight books. He also worked as a consultant psychiatrist until his retirement in 2014.

As well as being a devoted husband, father and grandfather, Peter had many talents and interests outside psychiatry and academia. He was a proficient horseman and accomplished guitarist. Following his retirement, he learned Welsh and became increasingly interested in musical composition. The world premiere of a string quartet which he wrote last year will take place at the Behavior Genetics Association meeting in June 2024, played by the Heath Quartet.

He was elected Fellow of the Royal College of Physicians of London in 1988 and Fellow of the Royal College of Psychiatrists in 1989. He was elected as a founding Fellow of the Academy of Medical Sciences in 1998 and served on its council. His other honours included Lifetime Achievement Awards from the International Society for Psychiatric Genetics (2007), King's College London (2012) and the Behavior Genetics Association (2023) and an Honorary Fellowship from Cardiff University (2008). He was appointed Commander of the Order of the British Empire in the 2016 Birthday Honours for services to biomedical research and psychiatric genetics.

He is survived by Anne, their three children Catrina, Liam and Lucy, and five grandchildren.

*This obituary is based on that published in* The Guardian *on 14 March 2024*.

